# Efficient Anchoring of *Erianthus* *arundinaceus* Chromatin Introgressed into Sugarcane by Specific Molecular Markers

**DOI:** 10.3390/ijms23169435

**Published:** 2022-08-21

**Authors:** Jiayun Wu, Mingxiao Zhang, Jiarui Liu, Yongji Huang, Liangnian Xu, Zuhu Deng, Xinwang Zhao

**Affiliations:** 1 National Engineering Research Center for Sugarcane, Fujian Agriculture and Forestry University, Fuzhou 350002, China; 2 Guangdong Sugarcane Genetic Improvement Engineering Center, Institute of Nanfan and Seed Industry, Guangdong Academy of Sciences, Guangzhou 510316, China; 3 State Key Laboratory for Protection and Utilization of Subtropical Agro-Bioresources, Guangxi University, Nanning 530004, China; 4 Key Lab of Sugarcane Biology and Genetic Breeding, Ministry of Agriculture, Fujian Agriculture and Forestry University, Fuzhou 350002, China

**Keywords:** sugarcane, *Erianthus* *arundinaceus*, species-specific molecular marker, chromosome, suppression subtractive hybridization (SSH), fluorescence in situ hybridization (FISH)

## Abstract

*Erianthus* *arundinaceus* is a valuable gene reservoir for sugarcane improvement. However, insufficient molecular markers for high-accuracy identification and tracking of the introgression status of *E.* *arundinaceus* chromatin impede sugarcane breeding. Fortunately, suppression subtractive hybridization (SSH) technology provides an excellent opportunity for the development of high-throughput *E.* *arundinaceus*-specific molecular markers at a reasonable cost. In this study, we constructed a SSH library of *E.* *arundinaceus*. In total, 288 clones of *E.* *arundinaceus*-specific repetitive sequences were screened out and their distribution patterns on chromosomes were characterized by fluorescence in situ hybridization (FISH). A subtelomeric repetitive sequence Ea086 and a diffusive repetitive sequence Ea009, plus 45S rDNA-bearing *E.* *arundinaceus* chromosome repetitive sequence EaITS were developed as *E.* *arundinaceus*-specific molecular markers, namely, Ea086-128, Ea009-257, and EaITS-278, covering all the *E.* *arundinaceus* chromosomes for high-accuracy identification of putative progeny. Both Ea086-128 and Ea009-257 were successfully applied to identify the authenticity of F_1_, BC_1_, BC_2_, BC_3_, and BC_4_ progeny between sugarcane and *E.* *arundinaceus*. In addition, EaITS-278 was a 45S rDNA-bearing *E.* *arundinaceus* chromosome-specific molecular marker for rapid tracking of the inherited status of this chromosome in a sugarcane background. Three BC_3_ progeny had apparently lost the 45S rDNA-bearing *E.* *arundinaceus* chromosome. We reported herein a highly effective and reliable SSH-based technology for discovery of high-throughput *E.* *arundinaceus*-specific sequences bearing high potential as molecular markers. Given its reliability and savings in time and efforts, the method is also suitable for development of species-specific molecular markers for other important wild relatives to accelerate introgression of wild relatives into sugarcane.

## 1. Introduction

In the past, the insatiable appetite for non-renewable fossil fuels has resulted in global climate change and environmental pollution [1]. To mitigate threats to human beings and the environment, greater use of renewable new energy sources could be the solution for environmentally sustainable economic growth in the long run [2]. In light of the abundance of sugar and lignocellulosic materials, sugarcane has been used as the most important sugar-producing crop and a renewable substrate source for biofuels [3]. Due to interspecific hybridization involving the frequent utilization of a limited number of parental clones, this has resulted in a narrow genetic base of modern sugarcane cultivars and limited resilience to biotic and abiotic stresses [4]. Thus, it has become an urgent task to remedy the growing concern of a dearth of genetic variation for sugarcane breeders. To broaden genetic diversity for increased productivity and better adaptability to a wide large range of growing conditions as well as providing more robust disease resistance, it is one efficient method, harnessing the beneficial genes of the wild relatives in sugarcane breeding. As one of the most important wild relatives of sugarcane, *Erianthus arundinaceus* has important potential for sugarcane breeding, such as conferring a strong root system, good ratooning ability and more generally, resistance to biotic and abiotic stresses [5]. So far, a series of genuine progeny have been produced in different backcrossed generations [5]. In future, a series of putative hybrid progeny will be selected as parents in sugarcane breeding programs, but recently, there has been no enough efficient molecular verification of putative hybrids. Obviously, it has become increasingly important to develop adequate molecular markers covering the whole *E. arundinaceus* genome for identification of progeny.

Traditionally, cytological methods and molecular markers are widely used to specifically detect the alien chromosomes and chromosomal segments in putative progeny. Genomic in situ hybridization (GISH) is a powerful cytological tool for identifying the introgression status of alien chromosomes in sugarcane background [5,6]. GISH results of progeny between sugarcane and *E. arundinaceus* indicated that chromosome transmission was n + n in F_1_, BC_2_, and BC_3_ generations, but was 2 n+ n in BC_1_ generation [4,5]. In addition, chromosome recombination between sugarcane and *E. arundinaceus* has also been characterized in BC_1_, BC_2_, and BC_3_ generations [4]. The major problem in developing the introgressions from *E. arundinaceus* into sugarcane is the selection of recombinants, although chromosome recombination between sugarcane and *E. arundinaceus* occurs only at low frequency. However, it is difficult to identify small segmental recombinants only using GISH. Furthermore, fluorescence in situ hybridization (FISH) is another cytological method involving the use of species-specific probes to detect alien chromosomes or chromosomal segments. In particular, the physical location of repetitive sequences by FISH, residing in prominent chromosomal positions, provides informative cytogenetic landmarks for unequivocal alien chromosome identification in many plant species. For example, FISH localized an *E. arundinaceus*-specific satellite DNA sequence in subtelomeric regions at one or both ends of most of the *E. arundinaceus* chromosomes. This *E. arundinaceus*-specific probe also had been used for the identification of genuine progeny between sugarcane and *E. arundinaceus* [7]. However, the potential of FISH to identify *E. arundinaceus* chromosomes and chromosomal segments is restricted by a limited number of suitable probes, low throughout, and ans inability to detect very small introgressions. Moreover, both FISH and GISH are time-consuming and need specific expertise and equipment [8].

Recent advances in molecular biology have made PCR-based markers a straightforward, affordable technique for rapid identification of putative progeny. Over several decades, molecular markers had been developed by conventional methods such as random amplified polymorphic DNA (RAPD), restriction fragment length polymorphism (RFLP), amplified fragment length polymorphism (AFLP), simple sequence repeats (SSR), inter simple sequence repeat (ISSR), and single nucleotide polymorphism (SNP) [9,10,11,12,13,14]. However, only limited molecular markers were used for identification of putative progeny between sugarcane and *E. arundinaceus*. For instance, Govindaraj et al. reported that fifteen specific sequenced tagged microsatellite site (STMS) markers from the sugarcane genome were successfully used in identifying four progeny between *S. spontaneum* and *E. arundinaceus* [14]. Cai et al. confirmed that two STMS markers and one 5S rDNA sequence-tagged marker allowed the identification of genuine intergeneric progeny in F_1_ and BC_1_ generations [15]. Moreover, Alix et al. isolated four *E. arundinaceus*-specific repetitive sequences by inter-*Alu*-like sequence-tagged PCR, which provide useful molecular markers for monitoring *E. arundinaceus* chromatin in sugarcane breeding programs [13]. Yang et al. used the probe from *E. arundinaceus*-specific repetitive sequence to identify *E. arundinaceus* chromatin in introgression progeny, but the method relied on cytological techniques. Nevertheless, these conventional methods used for development of specific molecular markers are time-consuming, laborious, and expensive [16]. In recent years, sequencing-based methods have also been able to greatly facilitate the availability of species-specific molecular markers, such as genotyping by sequencing (GBS) and the diversity arrays technology sequencing (DarT-seq), which were benefits for some grass species such as maize, wheat, and rice [17,18,19]. Nevertheless, developing species-specific molecular markers by using genomic tools has been relatively expensive. Additionally, the genome of sugarcane and its related species is complex due to the nature of polyploids [20], and such a highly complex genome poses challenges for developing species-specific molecular markers. Therefore, there is an urgent need to seek a high-throughput, reliable, and cost-effective method for the development of species-specific molecular markers to track *E. arundinaceus* chromosomes or chromosomal fragments.

Fortunately, suppressive subtractive hybridization (SSH) provides a high-throughput, high-accuracy, and low-cost tool for separating DNA sequences that distinguish two closely related genomic DNA (gDNA) [21,22,23,24]. This method does not require an in-depth knowledge of the genome and can thus be applied easily to non-model species, even the polyploids with a complex genome [22,24]. Indeed, SSH is a combination of normalization which equalizes the abundance of DNA fragments in the target species and subtraction which excludes sequences common to both the tester and the driver [21,25]. Thus, it is plausible that SSH will be developed for thousands of specific molecular markers in different species, enabling fast and reliable identification of putative progeny. In fact, this method had been proven to provide valuable insights into gDNA subtraction between different species. For instance, Li et al. reported that 617 species-specific DNA fragments were generated by SSH, and they could be used as a probe for the diagnosis of five species of the genus *Dendrobrium* [21]. Ge et al. demonstrated that 36 *Lophopyrum elongatum*-specific molecular markers were developed by SSH, which were successfully applicable in the detection of *L. elongatum* chromosomes or chromosomal fragments in wheat background [26]. In this study, the SSH technology was applied for development of *E. arundinaceus*-specific molecular markers. Development of *E. arundinaceus*-specific molecular markers will make it more convenient to identify *E. arundinaceus* chromatin from sugarcane background in introgression progeny and greatly improve the efficiency of *E. arundinaceus* introgressions into sugarcane. In future, the method is also suitable for development of species-specific molecular markers for other important wild relatives to accelerate introgression of wild relatives into sugarcane.

## 2. Results

### 2.1. Construction of an E. arundinaceus-Derived SSH Library

**Figure 1:**
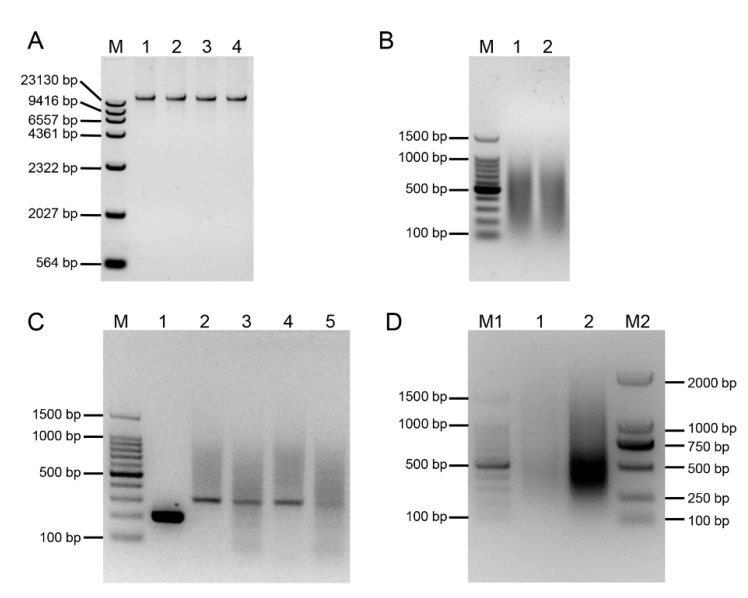
The electrophoresis result of an *E. arundinaceus*-derived SSH Library. (**A**) The electrophoresis result of gDNA. M: λDNA/HindIII digest DNA Marker; 1: *E. arundinaceus* HN92-77; 2: *S. officinarum* Badila; 3: *S. robustum* 51NG3; 4: *S. spontaneum* YN82-114. (**B**) Enzyme digestion of gDNA. M: 100 bp DNA Ladder; 1: The digested product of gDNA from *E. arundinaceus* HN92-77; 2: The digested product of gDNA from *S. officinarum* Badila, *S. robustum* 51NG3, and *S. spontaneum* YN82-114 by mixing equal quantity. (**C**) Detection result of the adaptor ligation efficiency. M: 100 bp DNA Ladder; 1: Amplification with primer pair 28S-204-F/28S-204-R by using tester with adaptor 1-ligated and adaptor 2R-ligated; 2: Amplification with primer pair PCR primer 1/28S-204-F by using tester with adaptor 1-ligated; 3: Amplification with primer pair PCR primer 1/28S-204-R by using tester with adaptor 1-ligated; 4: Amplification with primer pair PCR primer 1/28S-204-F by using tester with adaptor 2R-ligated; 5: Amplification with primer pair PCR primer 1/28S-204-R by using tester with adaptor 2R-ligated. (**D**) Detection result of two suppression PCR. M1: 100 bp DNA Ladder; 1: Primary PCR was performed with primer 1; 2: Secondary PCR was performed with both Nest primer 1 and Nest primer 2; M2: D2000 DNA Marker.

To develop the *E. arundinaceus*-specific molecular markers, an SSH library was constructed to eliminate the homologous sequences between *E. arundinaceus* and sugarcane and enrich the DNA unique to *E.* arundinaceus. Total gDNA was isolated and appeared undegraded on 1% agarose gel (Figure 1A). When double digested with both *Hae*III and *Alu*I, the tester and driver samples appeared as a smear between 0.1 to 2 kb in size (Figure 1B).

To analyze the adaptor ligation efficiency with the digested gDNA, the 28S rDNA primer pair (28S-204F/R) was designed by 28S rRNA gene as a control. A clear and bright band of 204 bp in size was amplified by PCR amplification using this primer pair. In addition, four different primer combinations were used to detect the amplify fragments that span the adaptor/DNA junctions of the tester gDNA fragments. The dim band of 300 bp in size was amplified by PCR amplification using these primer combinations (Figure 1C). Notably, the band intensity for these PCR products was four-fold greater than that of the control tester gDNA fragments, suggesting that at least 25% of the tester gDNA fragments have adaptors on both ends and the adaptor ligation with the gDNA was successful.

Primary PCR and secondary nested PCR of the tester gDNA fragments were performed after two rounds of subtraction hybridization between testers and drivers. The observable PCR products in primary PCR and secondary nested PCR appeared as a diffuse band between 100 and 1000 bp (Figure 1D). Additionally, the intensity of PCR products in secondary nested PCR was markedly greater than that in primary PCR. The difference in the amplification patterns between primary PCR and secondary nested PCR indicates a successful subtraction.

### 2.2. PCR Amplification and Dot-Blot Screening of E. arundinaceus-Specific Clones

**Figure 2:**
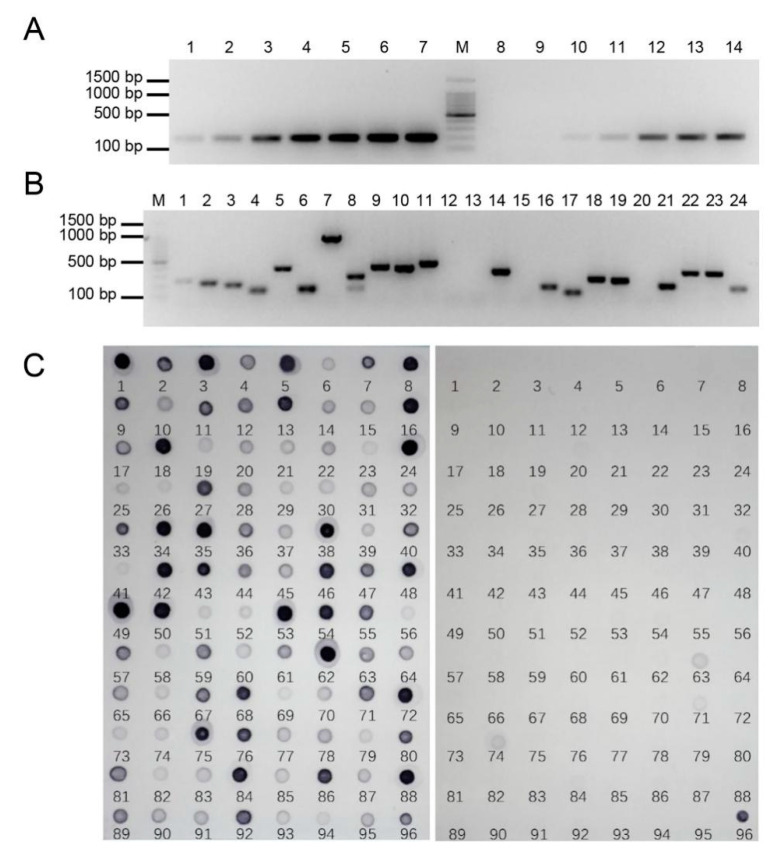
The detection of PCR and dot-blot screening of *E. arundinaceus*-specific clones. (**A**) The efficiency of suppression subtractive hybridization. M: 100bp Marker; 1–7: PCR was performed on unsubtracted secondary PCR products with amplification cycles of 18, 21, 24, 27, 30, 33, and 36; 8–14: PCR was performed on subtracted secondary PCR products with amplification cycles of 18, 21, 24, 27, 30, 33, and 36. (**B**) The detection result of positive clones. M: 100 bp DNA Ladder; 1–24: Randomly selected clones. (**C**) The detection result of RDB. The probe of gDNA from HN92-77 and sugarcane in left and right nylon membrane, respectively. The plasmid of 45S rDNA as positive control in No. 96.

To evaluate the subtraction efficiency after two rounds of subtraction, we detected the 28S rDNA in both the subtracted and non-subtracted gDNA pools using PCR amplification. If the abundance of the conserved 28S rDNA in the subtracted library was markedly reduced compared with the unsubtracted tester control, the subtraction would be efficient. As shown in Figure 2A, the 28S rDNA fragment in the unsubtracted tester was clearly visible after 18 cycles of amplification, while 24 cycles of amplification were required in the subtracted library. The abundance of 28S rDNA was effectively reduced, which indicated that gDNA homologous to both the tester and the driver had been highly subtracted.

A total of 400 clones were obtained from the SSH library. To eliminate the false positive clones as many as possible, the nest primer set 1/2R instead of the M13F/R primer set was used to amplify the selected clones, so that the recombinant clones were detected by PCR amplification. Most clone inserts ranged from 100 to 1000 bp, and the clones with two bands or no band were excluded (Figure 2B). In total, 329 positive clones were obtained from a subtractive library of *E. arundinaceus*, and the ratio of positive clones was 82.3%. This result verified that the clones were incorporated with high efficiency.

RDB was performed to screen out the *E. arundinaceus*-specific clones by hybridization with *E. arundinaceus* and sugarcane gDNA. As predicted, similar and obvious intensity of hybridization signals was observed for the 45S rDNA positive controls. However, differences in the intensity of hybridization signals were observed for most clones, suggesting these clones had different copy numbers (Figure 2C). A total of 288 clones, showing stronger signals when hybridized with *E. arundinaceus* gDNA but having no or weaker signals with the sugarcane gDNA, were identified and sequenced. Thus, the results showed that the *E. arundinaceus*-specific clones we obtained have high specificity.

### 2.3. Screening and Characterization of E. arundinaceus-Specific Clones Using FISH

**Figure 3:**
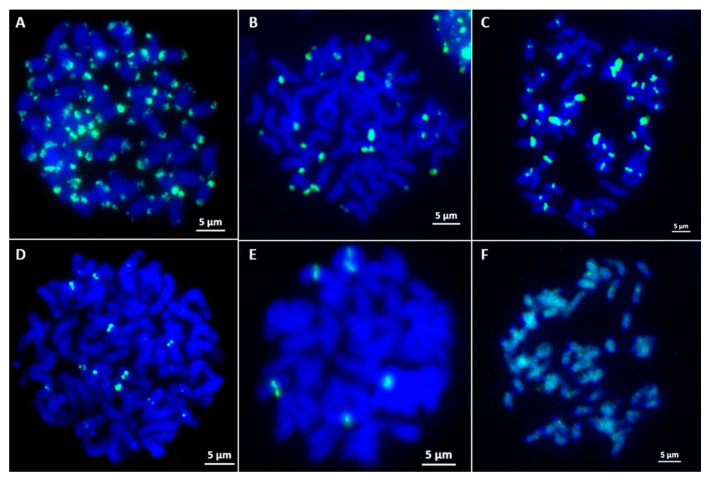
The FISH result of clones from SSH library. (**A**) The ends of most *E. arundinaceus* chromosomes; (**B**) The ends of a part of *E. arundinaceus* chromosomes; (**C**) The centromeric region of most *E. arundinaceus* chromosomes; (**D**) The centromeric region of a part of *E. arundinaceus* chromosomes; (**E**) The centromeric region of six *E. arundinaceus* chromosomes; (**F**) Diffuse distribute on all *E. arundinaceus* chromosomes, except for the ends of *E. arundinaceus* chromosomes. Scale bars = 5 μm.

To screen *E. arundinaceus*-specific chromosome markers, a total of 100 unique clones were randomly picked in the SSH library and examined by FISH on metaphase chromosomes of *E. arundinaceus* HN92-77 and HN92-105. The results showed that 86 clones produced distinct ‘dot’ or ‘block’ hybridization signals on the chromosomes. Among them, 49 clones (Ea001–Ea049) showed hybridization signals on the subtelomeric or telomeric regions on both arm ends on most of the *E. arundinaceus* chromosomes (Figure 3A), 16 clones (Ea050–Ea065) showed hybridization signals on the subtelomeric or telomeric regions either on one arm end or both arm ends on some *E. arundinaceus* chromosomes (Figure 3B), 4 clones (Ea066–Ea069) hybridized to the centromere on most of the *E. arundinaceus* chromosomes (Figure 3C), 15 clones (Ea070–Ea084) showed hybridization signals at centromeric regions on some *E. arundinaceus* chromosomes (Figure 3D), clone Ea085 showed only six hybridization signals in the centromeric regions (Figure 3E), clone Ea086 showed dispersed localization on *E. arundinaceus* chromosomes (Figure 3F). The remaining 14 clones showed no any hybridization signals on all the *E. arundinaceus* chromosomes, and thus, were not used for the development of *E. arundinaceus*-specific molecular markers.

### 2.4. Development of Candidate E. arundinaceus-Specific Molecular Markers

**Figure 4:**
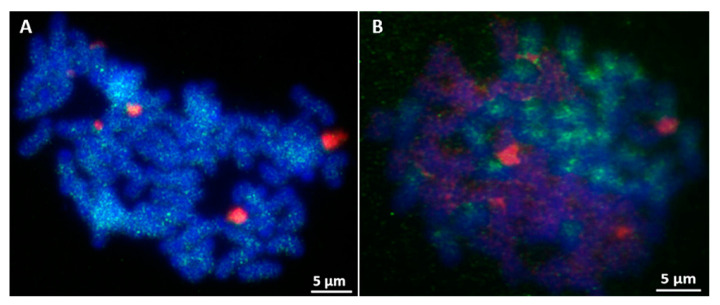
FISH result of clone Ea086 and 45S rDNA. (**A**) HN92-77; (**B**) YCE96-40. The probe of clone Ea086 was labelled with Dig (Green), the probes of both 45S rDNA and Badila gDNA were labelled with Biotin (Red), chromosomes were counterstained with DAPI (Blue). Scale bars = 5 μm.

**Figure 5:**
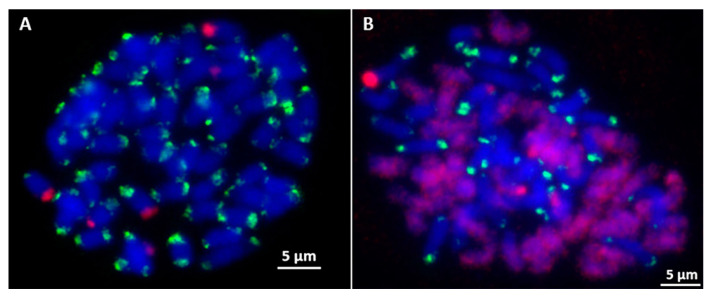
FISH result of clone Ea009 and 45S rDNA. (**A**) HN92-77; (**B**) YCE96-40. The probe of clone Ea009 was labelled with Dig (Green), the probes of both 45S rDNA and Badila gDNA were labelled with Biotin (Red), chromosomes were counterstained with DAPI (Blue). Scale bars = 5 μm.

FISH with Ea086 on *E. arundinaceus* mitotic chromosome spreads confirmed it was a highly dispersed repeat sequence over the length of every *E. arundinaceus* chromosomes but with fewer, less intense signals on both ends of the *E. arundinaceus* chromosomes (Figure 3F). In addition, the signals of repetitive DNA Ea009 appeared at both ends of most *E. arundinaceus* chromosomes (Figure 3A). FISH results showed that both Ea086 and Ea009 were *E. arundinaceus*-specific in *E. arundinaceus* HN92-77 and its F_1_ progeny YCE96-40 (Figure 4A,B and Figure 5A,B). Notably, six 45S rDNA loci were located at the terminal of six *E. arundinaceus* chromosomes (Figure 4A and Figure 5A). Therefore, we speculated that 45S rDNA might be located at that no localization signal on part of the chromosome. This speculation was confirmed by FISH on the metaphase cell chromosomes of *E. arundinaceus* HN92-77 and its F_1_ progeny YCE96-40 (Figure 5A,B). Totally, three candidate clones including Ea086, Ea009, and 45S rDNA were the reliable cytogenetic markers to unambiguously detect introgressions of *E. arundinaceus* chromatin in the sugarcane background. These three markers could be used for the development of *E. arundinaceus*-specific molecular markers. Therefore, clones Ea086 and Ea009 were sequenced and 45S rDNA ITS sequences were obtained on NCBI databases from sugarcane and *E. arundinaceus* for primer design. Depending on the size of amplification fragments, these three molecular markers were designated as Ea086-128, Ea009-257, and EaITS-278, respectively.

### 2.5. Validation of the Specificity and Stability of E. arundinaceus-Specific Markers

**Figure 6:**
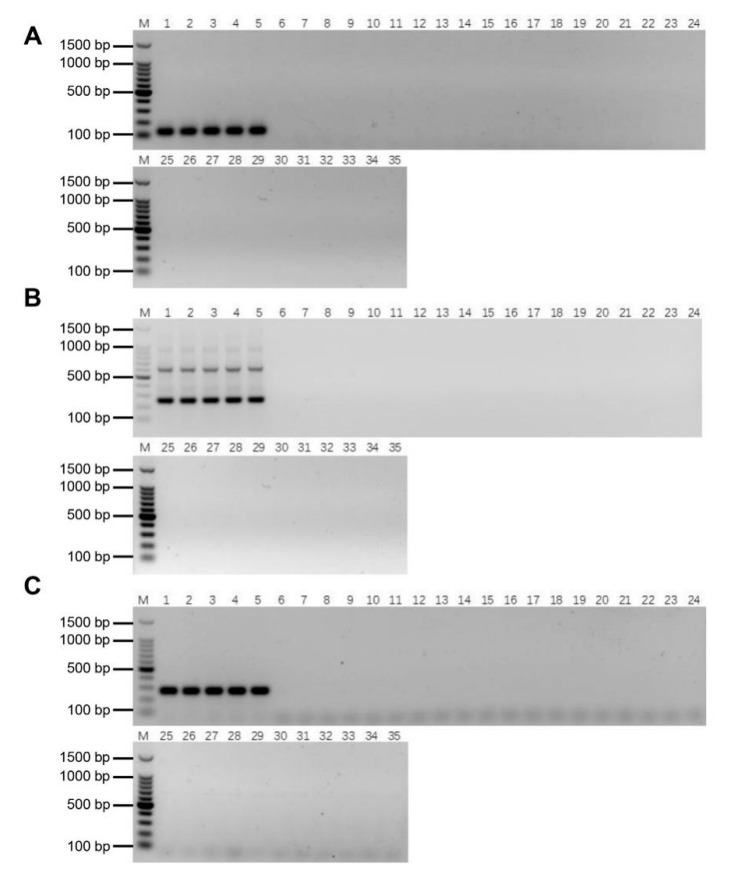
Validation of the specificity of *E. arundinaceus*-specific markers. (**A**) Ea086-128 primer; (**B**) Ea009-257 primer; (**C**) EaITS-278 primer. M: 100 bp DNA Ladder; 1–5: original species of *E. arundinaceus*; 6–10: *S. officinarum*; 11–15: *S. robustum*; 16–20: *S. spontaneum*; 21–25: *S. sinense*; 26–30: *S. barberi*; 31–35: Cultivars. All samples were listed in Table S1.

To confirm the specificity of *E. arundinaceus* specific markers, these three primers were used to amplify DNA in *E. arundinaceus*, *S. officinarum*, *S. robustum*, *S. spontaneum*, *S. sinense*, *S. barberi*, and cultivars, respectively. Specific bands could be amplified by these markers in gDNAs of five *E. arundinaceus* but not in that of the other species without *E. arundinaceus* chromatin (Figure 6A–C). It turned out that the *E. arundinaceus*-specific markers could be used for authenticity identification of progeny carrying *E. arundinaceus* chromatin. Notably, the specific primers Ea009-257 were able to amplify two distinct bands (Figure 6B), and then, the dimers of the repeat were sequenced to confirm the truth of the head-to-tail organization of the repeat.

**Figure 7:**
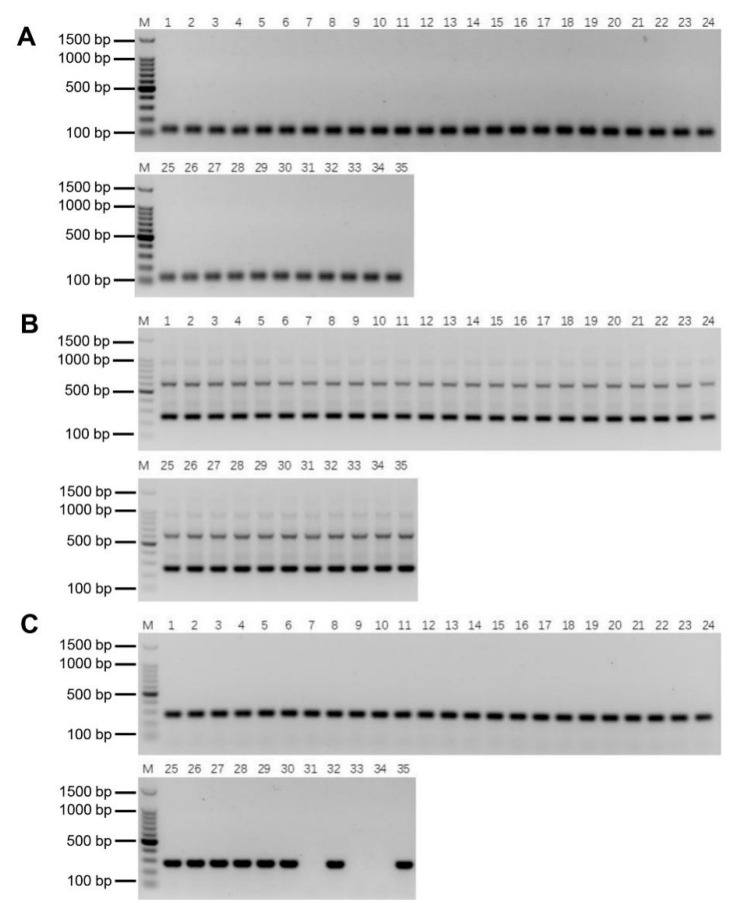
Validation of the stability of *E. arundinaceus*-specific markers. (**A**) Ea086-128 primer; (**B**) Ea009-257 primer; (**C**) EaITS-278 primer. M: 100 bp DNA Ladder; 1–5: F_1_ progeny between sugarcane and *E. arundinaceus*; 6–18: BC_1_ progeny between sugarcane and *E. arundinaceus*; 19–27: BC_2_ progeny between sugarcane and *E. arundinaceus*; 28–35: BC_3_ progeny between sugarcane and *E. arundinaceus*. All samples were listed in Table S2.

To confirm the stability of these molecular markers, PCR detection was performed in the F_1_, BC_1_, BC_2_, and BC_3_ progeny bearing *E. arundinaceus* chromatin as determined by GISH [4,27]. Both Ea086-128 and Ea009-257 amplified specific bands in all the progeny (Figure 7A–C), indicating that these two specific molecular markers of *E. arundinaceus* have good stability. EaITS-278 could amplify a specific band in most progeny except for YCE06-61, YCE06-111, and YCE06-140 (Figure 7C), indicating that the *E. arundinaceus*-derived chromosome carrying 45S rDNA in these three clones has been eliminated. Therefore, EaITS-278 can be used as a chromosome-specific marker to permit the tracking of the *E. arundinaceus*-derived chromosome carrying 45S rDNA. These results demonstrated that the three *E. arundinaceus*-specific markers screened could be reliably used to identify *E. arundinaceus* chromatin or the *E. arundinaceus*-derived chromosome carrying 45S rDNA.

### 2.6. Authenticity of the Putative BC_4_ Progeny

**Figure 8:**
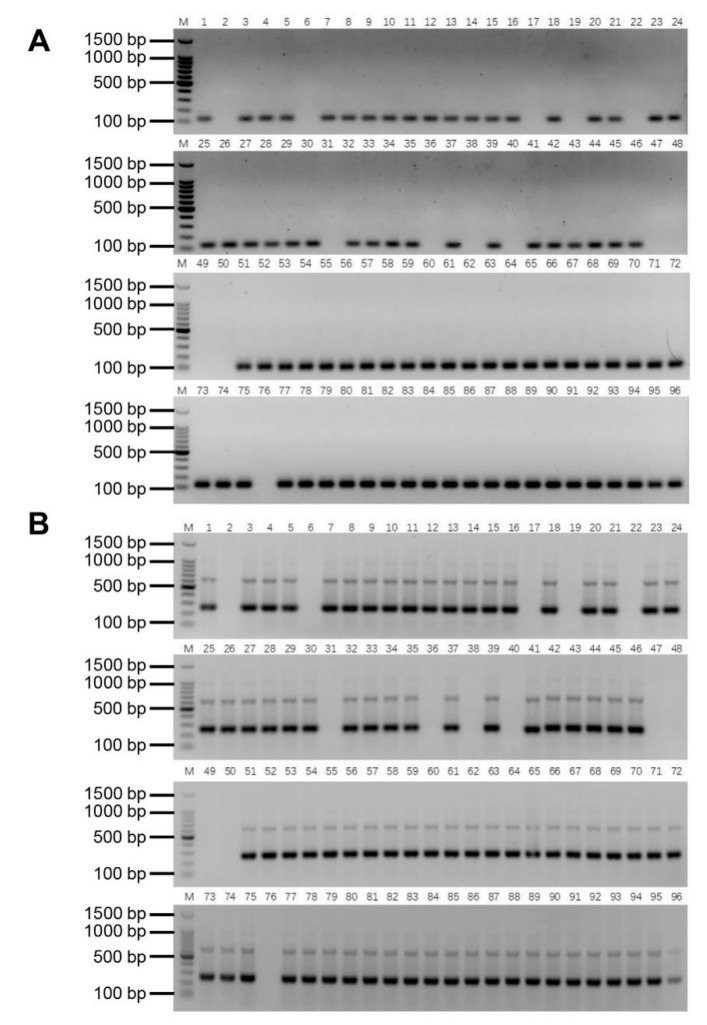
PCR detection result of putative BC_4_ progeny between sugarcane and *E. arundinaceus*. (**A**) Ea086-128 primer; (**B**) Ea009-257 primer. M: 100 bp DNA Ladder; 1–24: Putative progeny between CP89-2143 and YCE06-61; 25–48: Putative progeny between CP94-1100 and YCE06-61; 49–72: Putative progeny between HoCP01-564 and YCE06-61; 73–96: Putative progeny between GT00-122 and YCE06-61.

In order to gain insight on the introgression status of the *E. arundinaceus* chromatin into sugarcane in putative BC_4_ generation, a further investigation of 96 putative BC_4_ progeny from four different cross combinations was carried out (Table S3). YCE06-61, a BC_3_ progeny, was male parent with the *E. arundinaceus* lineages, and four sugarcane cultivars were female parents without the *E. arundinaceus* lineages. Given the elimination of the 45S rDNA-bearing chromosome in YCE06-61, it is no longer necessary to use EaITS-278 for PCR detection in the putative BC_4_ progeny. We used the *E. arundinaceus*-specific molecular markers Ea009-257 and Ea086-128 to verify the authenticity of the putative BC_4_ progeny in these cross combinations. Similar PCR results were obtained with these two specific molecular markers (Figure 8A,B). Among them, the rate of genuine progeny of crosses was 79.2% (CP89-2143 × YCE06-61) and 75.0% (CP94-1100 × YCE06-61), respectively, while the genuine progeny was identified with the higher rate up to 91.7% (HoCP01-564 × YCE06-61) and 95.8% (GT 00-122 × YCE06-61), respectively.

## 3. Discussion

*E. arundinaceus*, a wild relative species of the genus *Saccharum*, contains a largely untapped reservoir of agronomically important genes for sugarcane breeding. Currently, it is inevitable that utilizing introgressive hybridization to expand the genetic base of sugarcane has been identified. Hence, sugarcane breeders had implemented wide hybridization between sugarcane and *E. arundinaceus*, despite the large genetic distance between sugarcane and *E. arundinaceus*. More happily, subsequent introgression generations had also been obtained in sugarcane breeding programs [4,6,14,15,28]. Selecting introgression progeny with the best performance as good candidates is of vital importance for introgression breeding. It is evident that the availability of adequate *E. arundinaceus*-specific molecular markers can accelerate introgression breeding. However, the achievements are still not satisfactory, mostly resulting from a lack of *E. arundinaceus*-specific molecular markers. Therefore, there is an urgent need for development of *E. arundinaceus*-specific molecular markers to fulfill the demand for accurate and fast identification of putative progeny.

In the past four decades, several generic DNA fingerprinting methods, such as RAPD, RFLP, AFLP, SSR, ISSR, SNP, have been used in marker development for molecular plant breeding [9,10,11,12,13,14]. However, these methods are effective, but are labor-intensive and time-consuming. In our study, the PCR-based approach of utilizing *E. arundinaceus*-specific molecular markers to characterize introgression progeny is faster than FISH and GISH, which are traditionally employed for this purpose. Thus, sugarcane breeders are starving for development of *E. arundinaceus*-specific molecular markers for identification of the presence of the *E. arundinaceus* chromatin in putative progeny. Compared with conventional methods, SSH offers a simple, rapid, and affordable high-throughput screening method for separating DNA sequences that distinguish two closely related species. Although SSH only has been applied successfully to separate species-specific sequences in a few plants, this method had already proved to be capable of screening out a reservoir of species-specific molecular markers [21,26,28]. Notably, accurate identification of putative progeny hinges on the adequate molecular markers covering the whole genome. In the SSH array, the efficient amplification of species-specific sequences by PCR is thanks to those short and high copy number fragments tending to be amplified in preference to larger and lower copy number ones [29]. High-copy-number repetitive sequences comprise most eukaryotic genomes where they are major contributors to genome evolution [30,31]. Repetitive sequences can be species- or genome-specific, and even chromosome-specific in many species within a taxonomic family or diverse taxa, as some repetitive sequences are highly conserved while others are the evolutionarily fastest parts of the genome, showing pronounced differences even between closely related species [32]. Therefore, they form a reservoir of abundant DNA molecular markers covering the whole genome. Essentially, the SSH-based screening could reduce the screening task by removing a substantial number of the homologous sequences between different species and enriching the DNA sequences unique to the target species [21]. This is achieved by a combination of normalization which equalizes the abundance of DNA fragments within the target species, and subtraction which excludes sequences that are common to the two species being compared [21]. This significantly reduces the effort and increases the number of species-specific molecular markers. Our results demonstrated the utility of SSH for separation of the DNA sequences unique to *E. arundinaceus* and showed that SSH provides an easy, cheap, and conventional targeting species-compatible PCR screening method. Its real strength is that it offers a powerful and flexible tool for development of species-specific molecular markers in higher plants with a complex genome.

In this study, a unidirectional SSH library of *E. arundinaceus*-specific was constructed between *E. arundinaceus* and sugarcane. In total, 288 *E. arundinaceus*-specific sequences were obtained by SSH, suggesting that this SSH-based screening method could provide a highly parallel platform to efficiently obtain a larger number of species-specific sequences from a bulky and complex genome of target species. FISH screening trials of the SSH library resulted in the isolation of a large number of repetitive sequences located on all the chromosome end except on 45S rDNA, and a diffusive repetitive sequence on all chromosomes except the chromosome end, respectively (Figure 3A,F). Therefore, a subtelomeric repetitive sequence Ea086 and a diffusive repetitive sequence Ea009, plus 45S rDNA-bearing *E. arundinaceus* chromosome repetitive sequence EaITS, were developed as *E. arundinaceus*-specific molecular markers, covering all the *E. arundinaceus* chromosomes for high-accuracy identification of putative progeny. Hence, a large number of putative progeny could be easily and economically identified by PCR detection.

Consistent PCR detection results from both Ea086-128 and Ea009-257 were obtained in F_1_, BC_1_, BC_2_, and BC_3_ progeny between sugarcane and *E. arundinaceus*, and the PCR detection results determined are highly line with previous GISH results [4,27], validating that these two markers could be stably inherited in various progeny of different generations with *E. arundinaceus* chromatin (Figure 7). Consequently, these two markers were successfully applied to further identify the authenticity of 96 BC_4_ progeny between sugarcane and *E. arundinaceus*. These universal markers we developed are particularly valuable since they can be applied to easily track the transmission of *E. arundinaceus* chromosomes over generations, given the possibility of *E. arundinaceus* chromosome elimination in advanced generations. Furthermore, the developed molecular marker Ea009-257 was confirmed as a tandem repeat (Figure 6C). Many studies have shown that satellite DNAs comprising head-to-tail tandem repeats are believed to be the most dynamic components [33,34,35], undergoing the most rapid changes in the number and position of sites within a short evolutionary period [31]. It may also explain the largest number of *E. arundinaceus*-specific sequences from the SSH library. Noteworthy is that EaITS-278 was a 45S rDNA-bearing *E. arundinaceus* chromosome-specific molecular marker for rapid tracking of the inherited status of this chromosome in a sugarcane background. We found that three BC_3_ progeny, namely, YCE06-61, YCE06-111, and YCE06-140, had apparently lost the 45S rDNA-bearing *E. arundinaceus* chromosome. In the light of the GISH results, we could rule out that chromosome rearrangements between sugarcane and *E. arundinaceus* contribute to a loss or transposition of 45S rDNA sequences in this progeny. Taken together, these results demonstrated that the SSH-based technology is a highly effective and reliable approach for development of *E. arundinaceus*-specific molecular markers from a bulky and complex genome species. Given its reliability and savings in time and efforts, the method is also suitable for development of species-specific molecular markers for other important wild relatives to accelerate introgression of alien chromatin into sugarcane. Nevertheless, we do not recommend the replacement of GISH or FISH with molecular markers. Rather, it is highly encouraged to integrate efficient molecular markers with GISH or FISH strategies in sugarcane breeding programs to accelerate the process and improve outcomes. To be fair, we can not ignore the application of other new technology for development molecular markers. For instance, the next-generation sequencing (NGS) technology also provides a powerful tool for detecting large numbers of DNA markers within a short time-frame. Hence, application of any rapid and cost-effective approachs including NGS technology should be considered in development of molecular markers for other important wild relatives in future.

## 4. Materials and Methods

### 4.1. Plant Materials

Seven different types of accessions were used to test the specificity of primer, including *E. arundinaceus*, *Saccharum officinarum*, *S. robustum*, *S. spontaneum*, *S. sinense*, *S. barberi*, and cultivars (Table S1). Genuine progeny between sugarcane and *E. arundinaceus* identified by GISH were used for testing primer stability, including F_1_, BC_1_, BC_2_, and BC_3_ (Table S2). Putative BC_4_ progeny from four different cross combinations between YCE06-61 (BC_3_) as the male parent and different cultivars as the female parent were identified (Table S3). All the plant materials were provided by the Hainan Sugarcane Breeding Station, Guangzhou Sugarcane Industry Research Institute, as well as the Research Institute Ruili Station, the Sugarcane Research Institute of Yunnan Agriculture Science Academy.

### 4.2. Suppressive Subtractive Hybridization

SSH was carried out as described in instructions for the Clontech PCR-Select Bacterial Genome Subtraction Kit (Clontech, Mountain View, CA, USA). *E. arundinaceus* was assigned as the tester whereas *S. officinarum*, *S. robustum*, and *S. spontaneum* (gDNA pooled in 1:1:1 ratio) were assigned as the driver. Tester and driver gDNA were completely double digested at 37 °C for 2 h with both *Hae*III and *Alu*I (Promega, Madison, WI, USA) in a 50 μL reaction mixture. One hundred ng digested gDNA was ligated to 40 μM of adaptor 1and adaptor 2R. For evaluating the subtraction efficiency, 28S rDNA primer pairs (28S-F and 28S-R) were designed. Then, the adaptor-ligated tester (22.5 ng) underwent two rounds of hybridization with the excessive driver (225 ng) in a 4 μL reaction containing 1 × hybridization buffer. Two PCR amplifications were performed after subtraction. The first amplification was conducted in a 25 µL reaction that included 2 μL hybridization products, 1 × ExTaq buffer, 1.2 μM of P1 primer, 0.2 μM dNTP mixture, 1 U of ExTaq polymerase (TaKaRa ExTaq^TM^, Takara Biotechnology, Inc., Shiga, Japan). PCR was conducted using the following parameters: filling the adaptors for 8 min at 72 °C and denaturation for 2 min at 94 °C; 30 cycles of 95 °C for 30 s, 66 °C for 30 s, and 72 °C for 1 min, followed by a final extension at 72 °C for 5 min. One µL of the diluted tenfold PCR products was then amplified in secondary PCR in a 25 µL reaction containing 1 × PCR buffer, 1 µM nested PCR primer 1, 1.2 µM nested PCR primer 2R, 1 U of ExTaq polymerase, and 0.2 µM dNTP mixture, under the following cycling conditions: denaturation for 2 min at 94 °C; 30 cycles of 30 s at 94 °C, 30 s at 68 °C and 1 min at 72 °C, and a final extension for 5 min at 72 °C. The product of the second PCR was analyzed on 2% agarose gel stained with ethidium bromide. Secondary PCR products were purified by using a QIAquick PCR purification kit (Qiagen, Hilden, Germany) and ligated into the pMD19-T-vector (TaKaRa, Shiga, Japan). Plasmid DNA was purified using a Plasmid Mini kit I (OMEGA, Biel, Switzerland) and then quantified using a NanoVue Plus (GE Healthcare, Buckinghamshire, UK). All the primer sequences were provided in Table S4.

### 4.3. Preparation of Dig-Labeled gDNAs and Reverse Dot Blot (RDB)

Reverse dot blot (RDB) was performed as described by the instructions of the Digoxigenin (Dig) High Prime DNA Labeling and Detection Starter Kit I (Roche, Indianapolis, IN, USA) with slight modifications. The Dig-labeled gDNA of *E. arundinaceus* and sugarcane by nick translation was used to detect *E. arundinaceus*-specific clones. Aliquots of 40 ng of plasmids were denatured at 100 °C for 5 min and quickly chilled in an ice/water bath for 10 min, then were dotted onto Amersham Hybond N^+^ nylon membranes (GE Healthcare, Life Sciences, Indianapolis, IN, USA). Altogether, two pairs of blots were prepared and subsequently cross-linked by using a Stratalinker^TM^ UV Crosslinker (Stratagene, LA Jolla, CA, USA). After crosslink, the membrane was prehybridized with hybridization buffer (6 × SSC, 5 × Denhardt’s, 0.5% SDS, and 100 μg/mL sheared salmon sperm DNA) at 42 °C for 30 min. Hybridizations were performed overnight at 42 °C in the hybridization buffer containing Dig-labeled probe. High stringency washes were performed following a rinse in wash solution containing 0.5 × saline-sodium citrate (SSC) and 0.1% sodium dodecyl sulfate at room temperature before the blots were washed twice at 68 °C for 15 min each. After incubation, each for 30 min in blocking solution and antibody solution, respectively, the membranes were washed for 15 min twice in washing buffer. Then, the membranes were equilibrated for 5 min in detection buffer and incubated for 6 h in color substrate solution in the dark. Finally, hybridization signals were detected with ChemiDocXRS (Bio-Rad, Hercules, CA, USA). The dot blot analysis was repeated thrice with three independent sets of blots, and the DNA of 45S rDNA plasmid was used as the positive control. A total of 288 *E. arundinaceus*-specific sequences were obtained and sequenced by Beijing Genomics Institute (BGI) Co., Ltd. (Shenzhen, China). Sequence data for these *E. arundinaceus*-specific sequences have been uploaded to the GenBank data library under accession numbers MN813187-MN813475.

### 4.4. Fluorescence In Situ Hybridization (FISH)

To prepare mitotic metaphase chromosomes, root tips were harvested from greenhouse-grown plants and treated with 2 mM 8-hydroxyquinoline at room temperature for 2 h. The root tips were then fixed in ethanol: acetic acid (3:1) fixative solution. The meristem regions were cut from root tips using a razor blade and incubated in an enzyme solution with 2% cellulase (Yakult Pharmaceutical, Tokyo, Japan) and 1% pectolyase (Sigma Chemical, St. Louis, MO, USA) at 37 °C for 2 h. The roots were mechanically disrupted with a metal pick and 10 μL of the solution dropped onto microscope slides. At least ten mitotic metaphase chromosomes from three different root tips were prepared from each clone.

FISH was carried out essentially as described previously by Kato et al. [36]. Prior to FISH, chromosomal DNA on slides was denatured in 70% formamide and 2 × SSC at 80 °C for 3 min followed by dehydration in 70, 90, and 100% ethanol each for 5 min at −20 °C. About 2 μg of sheared gDNA with average size of 100 bp prepared from *E. arundinaceus* was used as blocking DNA. The hybridization mixture (25 μL) containing 50 ng of probe, 10% dextran sulfate, 50% formamide, and 2 × SSC was denatured at 95 °C for 5 min prior to application to slides and hybridization overnight at 37 °C. Following by stringency washes (2 × SSC, 50% formamide in 2 × SSC, and at 42 °C in 2 × SSC for 5 min each), Dig-labeled probes were detected by sheep-anti-digoxin-FITC (Roche, Lewes, UK) and rabbit-anti-sheep-FITC (Roche, Lewes, UK), respectively; Biotin-labeled probes were detected by Avidin D, Rhodamine 600 (XRITC) and biotinylated anti-avidin antibody (Vector Laboratories, Burlingame, CA, USA), respectively. Chromosomes were counterstained with 4’, 6-diamidino-2-phenylindole (DAPI) in a Vectashield anti-fade solution (Vector Laboratories, Burlingame, CA, USA). FISH signals were captured using the AxioVision measurement module of an Axio Scope A1 Imager fluorescent microscope (Zeiss, Jena, Germany).

### 4.5. Development, Verification, and Detection of E. arundinaceus-Specific Molecular Markers

The multiple sequence alignment of 45S rDNA ITS sequences from *S. officinarum*, *S. robustum*, *S. spontaneum*, and *E. arundinaceus* are shown in Figure S1. The program Primer 5 was used to design oligonucleotide primers on the sequences of Ea086, Ea009, and EaITS. The primer sequences of Ea086, Ea009, and EaITS were provided in Table S4. PCR reactions were carried out in a final volume of 10 µL with 50 ng template DNA, 1 × ExTaq buffer, 0.2 μM dNTP mixture, 0.8 μM each primer, and 1 U of ExTaq polymerase (TaKaRa ExTaq^TM^, Takara Biotechnology Inc.). PCR amplification was conducted using the following procedure: PCR amplifications were performed at 94 °C for 5 min, 35 cycles of 30 s at 94 °C, 30 s at 56 °C for annealing, and 1 min of extension at 72 °C, ending with 5 min at 72 °C. Amplification products were visualized using 2% agarose gels.

## 5. Conclusions

In this study, the SSH technology is demonstrated to be a highly effective and reliable approach for development of *E. arundinaceus*-specific molecular markers. These molecular markers developed are very beneficial for progressive research in sugarcane breeding, particularly in the process of developing and characterizing introgression progeny. Additionally, a further analysis revealed remarkable stability of Ea086-128 and Ea009-257 in different generations, as they can be deployed to characterize introgression progeny carrying *E. arundinaceus* chromosomes. Hence, their high stability will further broaden their scope of application in putative BC_4_ generation and even more advanced generations. Additionally, EaITS-278 could be developed for rapid tracking the inherited status of the 45S rDNA-bearing *E. arundinaceus* chromosome in sugarcane background. Altogether, these findings indicate that integrating the markers with GISH or FISH strategies would accelerate development and characterization of introgression progeny. In future, the method is also suitable for development of species-specific molecular markers for other important wild relatives to accelerate introgression of wild relatives into sugarcane.

## Data Availability

Not applicable.

## Supplementary Materials

The following supporting information can be downloaded at: https://www.mdpi.com/article/10.3390/ijms23169435/s1.
