# Supplementary material for: Efficient Anchoring of Erianthus arundinaceus Chromatin Introgressed into Sugarcane by Specific Molecular Markers
Source: Int J Mol Sci. 2022 Aug 21;23(16):9435. doi: 10.3390/ijms23169435 (PMC9408830; doi:10.3390/ijms23169435)
Supplement: Supplementary file 1 [file ijms-23-09435-s001.zip › ijms-1881593-supplementary.pdf]

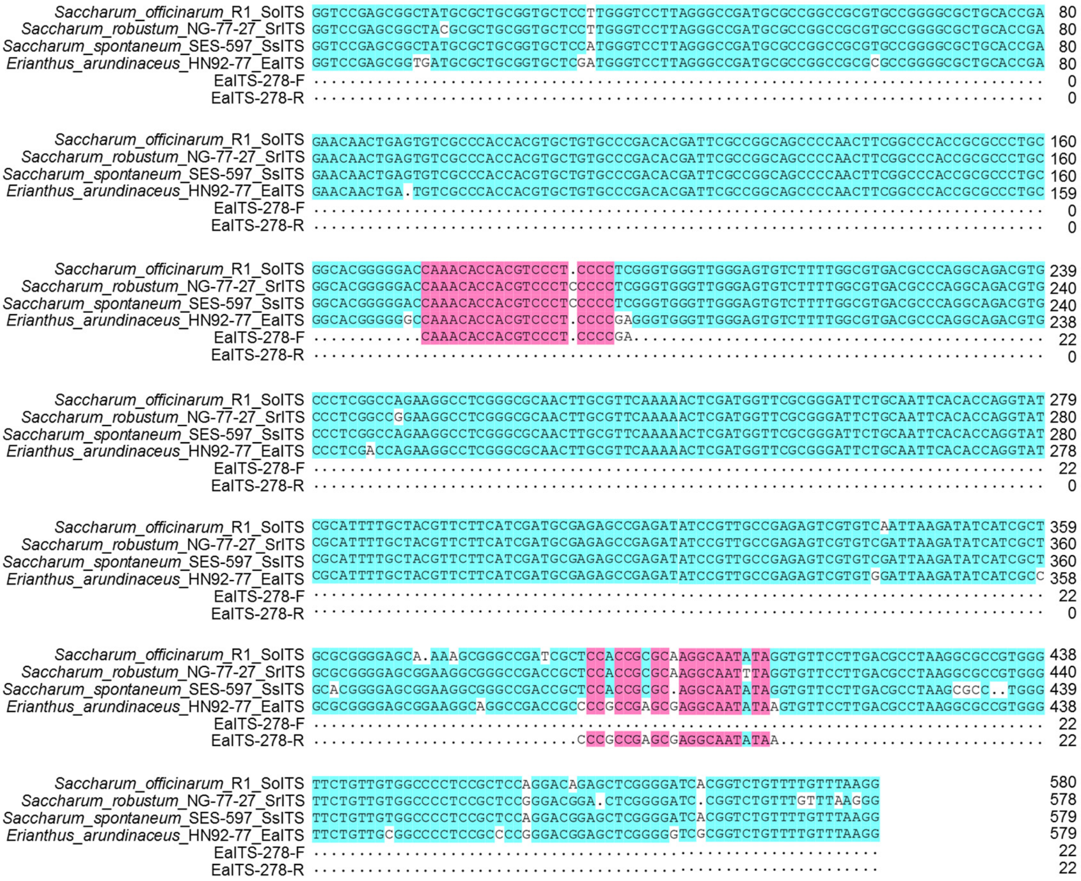

Figure S1. The multiple sequence alignment of 45S rDNA ITS sequences from *S. officinarum*, *S. robustum*, *S. spontaneum*, and *E. arundinaceus* as well as the position of the EaITS primer pairs (EaITS-278F and EaITS-278-R).

Table S1. Clones of *E. arundinaceus* and sugarcane.

No.	Clones	Species	Origin
1	HN92-77	*E. arundinaceus*	Hainan, China
2	HN92-105	*E. arundinaceus*	Hainan, China
3	YN82-30	*E. arundinaceus*	Yunnan, China
4	YN82-80	*E. arundinaceus*	Yunnan, China
5	YN83-180	*E. arundinaceus*	Yunnan, China
6	Badila	*S. officinarum*	New Guinea
7	Crystallina	*S. officinarum*	New Guinea
8	Black Cheribon	*S. officinarum*	New Guinea
9	Lothers	*S. officinarum*	New Guinea
10	Luohanzhe	*S. officinarum*	Guangxi, China
11	51NG63	*S. robustum*	New Guinea
12	NG77-004	*S. robustum*	New Guinea
13	57NG208	*S. robustum*	New Guinea
14	51NG3	*S. robustum*	New Guinea
15	Daye	*S. robustum*	Fujian, China
16	YN75-2-11	*S. spontaneum*	Yunnan, China
17	YN82-50	*S. spontaneum*	Yunnan, China
18	FJ92-1-11	*S. spontaneum*	Fujian, China
19	FJ89-1-18	*S. spontaneum*	Fujian, China
20	FJ89-1-19	*S. spontaneum*	Fujian, China
21	Yongshengzhe	*S. sinense*	Yunnan, China
22	Wenshanzhe	*S. sinense*	Yunnan, China
23	Uba	*S. sinense*	Guangdong, China
24	Tanzhou Zhuzhe	*S. sinense*	Guangdong, China
25	Guangxi Zhuzhe	*S. sinense*	Guangxi, China
26	Katha	*S. barberi*	India
27	Nagans	*S. barberi*	India
28	Pansahi	*S. barberi*	India
29	Saretha	*S. barberi*	India
30	Mungo	*S. barberi*	India
31	R570	Cultivars	Reunion island, Mauritius
32	ROC22	Cultivars	Taiwan, China
33	CP84-1198	Cultivars	Florida, USA
34	F172	Cultivars	Taiwan, China
35	ROC10	Cultivars	Taiwan, China

Table S2. Clones of F_1, BC_1, BC_2 and BC_3 between sugarcane and *E. arundinaceus.*

No.	Clones	Generation	Male parent	Female parent
1	YCE96-43	F_1	HN92-77	Badila
2	YCE96-45	F_1	HN92-77	Badila
3	YCE95-41	F_1	HN92-77	Badila
4	YCE96-66	F_1	HN92-105	Badila
5	YCE96-40	F_1	HN92-77	Badila
6	YCE01-33	BC_1	YCE95-41	CP84-1198
7	YCE01-46	BC_1	YCE95-41	CP84-1198
8	YCE01-48	BC_1	YCE95-41	CP84-1198
9	YCE01-63	BC_1	YCE96-66	CP84-1198
10	YCE01-99	BC_1	YCE96-40	CP84-1198
11	YCE01-102	BC_1	YCE96-40	CP84-1198
12	YCE01-105	BC_1	YCE96-40	CP84-1198
13	YCE01-116	BC_1	YCE96-40	CP84-1198
14	YCE01-134	BC_1	YCE96-40	CP84-1198
15	YCE01-36	BC_1	YCE96-40	CP84-1198
16	YCE01-92	BC_1	YCE96-40	CP84-1198
17	YCE01-61	BC_1	YCE96-66	CP84-1198
18	YCE01-69	BC_1	YCE96-66	CP84-1198
19	YCE03-01	BC_2	NJ57-416	YCE01-116
20	YCE03-06	BC_2	YCE01-116	NJ57-416
21	YCE03-16	BC_2	YCE01-91	ROC23
22	YCE03-168	BC_2	YCE01-123	ROC10
23	YCE03-218	BC_2	YT73-204	YCE01-105
24	YCE03-249	BC_2	YCE01-69	YT73-204
25	YCE03-378	BC_2	ROC20	YCE01-92
26	YCE04-55	BC_2	YC95-46	YCE01-102
27	YCE05-179	BC_2	ROC20	YCE01-134
28	YCE05-64	BC_3	YT73-204	YCE03-133
29	YCE05-150	BC_3	YCE03-218	ROC10
30	YCE06-63	BC_3	ROC10	YCE03-01
31	YCE06-61	BC_3	ROC10	YCE03-01
32	YCE06-92	BC_3	YCE04-51	YT93-159
33	YCE06-111	BC_3	YCE03-168	YT93-159
34	YCE06-140	BC_3	YCE03-218	ROC10
35	YCE06-166	BC_3	YCE03-168	YT91-976

Table S3. Putative BC_4 progeny between sugarcane and *E. arundinaceus*.

No.	Cross combination	Female parent	Male parent
1	CP89-2143 × YCE06-61	CP89-2143	YCE06-61
2	CP94-1100 × YCE06-61	CP94-1100	YCE06-61
3	HoCP01-564 × YCE06-61	HoCP01-564	YCE06-61
4	GT00-122 × YCE06-61	GT00-122	YCE06-61

Table S4. Primer sequence.

Primer	Sequence (5′-3′)	Length (bp)
adaptor 1	CTAATACGACTCACTATAGGGCTCGAGCGGCCGC CCGGGCAGAG	44
adaptor 2R	CTAATACGACTCACTATAGGGCAGCGTGGTCGCG GCCGAGAG	42
28S-F	GTGCATTAAGTGTGGAGA	18
28S-R	TAGGCTCCTTCTCGAGCTCT	20
primer 1	TCGAGCGGCCGCCCGGGCAGAG	22
primer 2R	AGCGTGGTCGCGGCCGAGAG	20
Ea086-128-F	TAGTAGTGCGTCGGGTCA	18
Ea086-128-R	CAAGGTTCGTGTTTATCT	18
Ea009-257-F	GAATTGGGAATAGCGGGACT	20
Ea009-257-R	CATCGGCTGTTCCTGGTAT	19
EaITS-278-F	CAAACACCACGTCCCTCCCCGA	22
EaITS-278-R	TTATATTGCCTCGCTCGGCGGG	22
